# Antenatal telephone support intervention with and without uterine artery Doppler screening for low risk nulliparous women: a randomised controlled trial

**DOI:** 10.1186/1471-2393-14-121

**Published:** 2014-03-31

**Authors:** Vikki J Snaith, Jenny Hewison, Ian N Steen, Stephen C Robson

**Affiliations:** 1 The Newcastle upon Tyne Hospitals NHS Foundation Trust, Newcastle upon Tyne, UK; 2 Leeds Institute of Health Sciences, University of Leeds, Leeds, UK; 3 Institute of Health and Society, Newcastle University, Newcastle upon Tyne, UK; 4 Institute of Cellular Medicine, Newcastle University, Newcastle upon Tyne, UK

**Keywords:** Antenatal care, Antenatal visits, Telephone support intervention, Uterine artery Doppler screening, Anxiety

## Abstract

**Background:**

The number of routine antenatal visits provided to low risk nulliparous women has been reduced in the UK, acknowledging this change in care may result in women being less satisfied with their care and having poorer psychosocial outcomes. The primary aim of the study was to investigate whether the provision of proactive telephone support intervention (TSI) with and without uterine artery Doppler screening (UADS) would reduce the total number of antenatal visits required. A secondary aim was to investigate whether the interventions affected psychological outcomes.

**Methods:**

A three-arm randomised controlled trial involving 840 low risk nulliparous women was conducted at a large maternity unit in North East England. All women received antenatal care in line with current UK guidance. Women in the TSI group (T) received calls from a midwife at 28, 33 and 36 weeks and women in the telephone and Doppler group (T + D) received the TSI and additional UADS at 20 weeks’ gestation. The main outcome measure was the total number of scheduled and unscheduled antenatal visits received after 20 weeks’ gestation.

**Results:**

The median number of unscheduled (n = 2.0), scheduled visits (n = 7.0) and mean number of total visits (n = 8.8) were similar in the three groups. The majority (67%) of additional antenatal visits were made to a Maternity Assessment Unit because of commonly occurring pregnancy complications. Additional TSI+/–UADS was not associated with differences in clinical outcomes, levels of anxiety, social support or satisfaction with care. There were challenges to the successful delivery of the telephone support intervention; 59% of women were contacted at 29 and 33 weeks gestation reducing to 52% of women at 37 weeks.

**Conclusions:**

Provision of additional telephone support (with or without UADS) in low risk nulliparous women did not reduce the number of unscheduled antenatal visits or reduce anxiety. This study provides a useful insight into the reasons why this client group attend for unscheduled visits.

**Trial registration:**

ISRCTN62354584

## Background

The scheduled number of antenatal visits offered to low risk nulliparous women in England and Wales was reduced to ten visits in 2003 [1]. Seven trials, including four conducted in developed countries, have addressed the impact of a policy of reduced antenatal visits [2]. Policy implementation proved challenging; women in the intervention arm of three of the five trials undertaken in developed countries had a greater number of antenatal visits than planned [3-5]. The systematic reviews of these studies concluded that a reduced visits schedule could be implemented without any adverse impact on maternal and perinatal outcomes [2] but this may result in women feeling less satisfied with their care [3,6].

Compared to multiparous women, nulliparous women have different needs and expectations of antenatal care provision; they place greater importance on gaining information and attending antenatal classes [7]. This may relate to greater pregnancy specific worries [8,9], specifically in relation to there being something wrong with the baby, caring for the baby and giving birth [10]. Indeed, Sikorski et al. reported that nulliparous women were more likely to decline participation in their trial of reduced visits, suggesting that they found the prospect of fewer visits to be less acceptable than multiparous women [3]. Thus the clinical and economic impact of a reduced schedule of visits in nulliparous women remains uncertain.

Although antenatal care has historically been provided by face to face visits, telephone contact has the potential to provide an alternative means of communication and support [11]. The increase in telephone-delivered interventions has occurred partly in response to a need to reduce health care costs [12]. In addition, access to a telephone is almost universal; therefore utilisation of this technology to deliver healthcare and support is a feasible, low-cost option [13] which reduces barriers to accessing healthcare such as transport and geographical challenges [14].

Telephone contact has been utilised to deliver a variety of antenatal and postnatal support interventions to breastfeeding mothers [15,16], smokers [12,17] and women at increased risk of low birthweight infants [18], postnatal depression [19]and preterm birth [20,21]. The results were variable with no improvement in birthweight, rates of preterm delivery or smoking but an increase in breastfeeding duration and reduction in depression scores.

In a previous study of a reduced antenatal visit schedule, 86% of participants stated that they would like extra telephone contact with a midwife as a means of maintaining support during pregnancy when visits were reduced [22]. Telephone support may provide a method of increasing adherence to and satisfaction with a reduced visit schedule.

Nulliparous women are increased risk of placental-mediated disorders, including preeclampsia (PE) and delivery of a small-for-gestational age (SGA) infant [23]. Uterine artery Doppler screening (UADS) provides a method of screening for placental-mediated disorders; abnormal flow velocities correlate with deficient spiral artery remodelling [24] and a review of 52 studies concluded that an increased second trimester pulsatility index (PI) with diastolic notching was a useful predictor of PE (positive likelihood ratio [LR+] 7.5, 95% CI 5.4-10.2); and SGA (LR + 9.1, 95% CI 5.0-16.7) in a low risk population [25]. Women who have normal UADS constitute a lower risk group for the development of PE (negative LR [LR-] 0.59, 95% CI 0.47-0.71) and SGA (LR- 0.89, 95% CI 0.85-0.93). Thus, providing nulliparous women with information about their individualised risk of developing PE and SGA may reduce their requirement for face-to-face visits with a midwife [26]. Previous findings have shown that pregnant women generally welcome the offer of screening tests during pregnancy, providing they are given appropriate information with which to make an informed choice [27,28].

In light of these findings, the present trial was designed to determine the impact of telephone support and UADS on the total number of antenatal visits in low risk nulliparous women receiving the recommended reduced schedule of care [26]. Total visits were perceived to be an important outcome given that prior trials in developed countries had failed to implement a reduced visit schedule [3,4,6]. Specifically the study was designed to test the hypotheses that provision of a telephone support intervention, with or without supplemental UADS at 20 weeks of pregnancy, would reduce the total number of antenatal visits (routine and additional visits), reduce anxiety and increase social support and satisfaction with antenatal care when compared with usual antenatal care [26].

## Methods

A three-arm randomised controlled trial was conducted in the Newcastle upon Tyne Hospitals NHS Foundation Trust between February 2004 and January 2007 (follow up completed in July 2007). The trial was approved by the Newcastle and North Tyneside Local Research Ethics Committee (Ref: 2003/208) and was registered on the International Standard Randomised Controlled Trial Number Register (http://ISRCTN62354584). Nulliparous women were suitable for inclusion if they met the definition of low risk as defined by the NICE guidelines [1].

Eligible women were approached to take part in the study by a research midwife when attending for their 20 week fetal anomaly scan. They were provided with an information sheet and full verbal explanation of the study was given. Following a decision to participate in the study, written consent was obtained and randomisation was undertaken by a web based randomisation package provided by the Centre for Health Services Research based at Newcastle University. Women who declined recruitment were asked to state their reason for not participating. Women who were unable to speak English and those who planned to relocate to a different geographical area during their pregnancy were excluded from the study. Teenage women who were receiving input from the hospital-based teenage pregnancy support team were also excluded.

Women randomised to the control group (C) were allocated to receive standard antenatal care, which comprised of ten routine antenatal visits (seven visits after 20 weeks’ gestation) [1]. Women randomised to the telephone support group (T) received the TSI in addition to usual care and were contacted by a designated midwife at 29, 33 and 37 weeks’ gestation (as determined from a first trimester ultrasound scan). Women were asked at the time of day they would prefer to be contacted. They were given the option of morning, afternoon or evening calls on any day of the week to accommodate work patterns and personal commitments. The midwife attempted to contact the women twice within the pre-arranged time frame; if both calls were unsuccessful this was recorded and calls attempted at the next gestational time point. If the woman reported concerns that the study midwife felt required further investigation by another health professional, an appropriate referral was made according to the hospital guidelines. The midwife also directed women to alternative sources of information such as online resources e.g. to obtain information about maternity benefits.

A vital function of the TSI was that it centred on the specific needs of the woman. Although the midwife initiated the dialogue, the aim was that it was subsequently driven by the concerns of the woman with the expectation that this would vary between individuals. To ensure consistency in the way in which the TSI was conducted, a discussion guide was developed which incorporated questions covering maternal physical health, availability of practical and emotional support, personal and fetal wellbeing. The midwife who delivered the intervention had experience in providing advice to pregnant women via the telephone as a result of working within the hospital Maternity Assessment Unit (MAU) and was given specific training about how to deliver the intervention.

The women who were randomised to the telephone support and Doppler (T + D) group had UADS performed at the end of the routine 20 week anomaly scan. The ultrasound examinations during the trial were undertaken on two machines: Aloka 5000 and Philips HD 11XE. Colour flow was used to identify the right and left uterine arteries at the crossover with the external iliac arteries and the sample volume was adjusted and positioned over each uterine artery. Pulsed wave Doppler was used to collect the waveforms [29]. The pulsatility index (PI) of each uterine artery was measured over five cycles (by the system software) and the mean of the left and right uterine artery PI was calculated manually [29]. The presence of unilateral or bilateral waveform notching was noted. This information, together with the 20 week scan findings, was recorded on the women’s record in the ultrasound database (ViewPoint, GE Healthcare). A normal uterine artery Doppler was defined as a mean uterine artery PI of <1.45 with no or unilateral notch [30]. Participants with normal UADS received explicit verbal and written information about their reduced risk of developing PE and of delivering a SGA baby.

Women with abnormal UADS (defined as a mean PI of ≥1.45 and/or bilateral notching [30]) were provided with a verbal explanation of the result and an information sheet detailing the possible implications and resulting pathway of care. This included a repeat ultrasound assessment at 24 weeks’ gestation. They were encouraged to contact a study midwife for further discussion if they had any concerns about the UADS result. If repeat UADS was normal at 24 weeks the women were informed of the result and given an information sheet about their reduced risk. The remainder of their antenatal care followed the usual care schedule supplemented by the TSI. If the UADS result remained abnormal an ultrasound assessment of fetal size, umbilical artery Doppler PI and amniotic fluid index was arranged for 30 weeks’ gestation. If any of the parameters measured were outside of the normal range follow up was arranged according to the hospital guidelines. The participants in this group received the TSI irrespective of the result of their UADS.

The primary outcome measure was the number of antenatal visits that women received after 20 weeks’ gestation. Antenatal visits were defined as a scheduled or unscheduled attendance at a medical, midwife or GP clinic within the community or hospital setting, attendance at a maternity assessment unit, home visit by a community midwife or general practitioner and attendance at any other location for the receipt of antenatal care such as the fetal medicine unit or hospital ward (including additional ultrasound scans). This was to ensure that the total number of visits made by participants incorporated all of the ‘face to face’ contacts women accessed, including those additional to the scheduled visits provided by their midwife. All antenatal contacts were included provided they were recorded in either the women’s hospital or handheld antenatal notes and irrespective of whether the contact was initiated by the woman, her midwife or other health professional.

All of the participants were asked to complete a questionnaire at 20, 28, 36 weeks’ gestation and six weeks’ post delivery. The first questionnaire was given at the time of recruitment (20 weeks’ gestation) and the other questionnaires were sent by post with a self addressed envelope for return. The following scales were used in the questionnaires.

The State-Trait Anxiety Inventory (STAI) [31] comprises of two 20 item scales; one scale is designed to measure trait anxiety and the other to measure state anxiety. Trait anxiety is described as the general level of anxiety experienced by an individual that determines how they react to perceived threatening situations. State anxiety reflects how the individual feels at the time the measure is completed [32]. The STAI scores range from 20 to 80 for both the trait and the state subscales with a higher score indicating higher levels of anxiety [31]. Both subscales were measured at recruitment to obtain a baseline assessment of women’s anxiety. Subsequent questionnaires at 28, 36 and 6 weeks postnatal included only the state subscale.

Levels of social support were measured using the Duke/UNC Functional Social support (DUFSS) questionnaire, designed to measure functional aspects of social support within a primary care setting [33]. Each question has five possible responses ranging from a score of one (corresponding to ‘as much as I would like’) to a score of five (corresponding to ‘much less than I would like’). Total scores range from 8 to 40 with a lower score indicating higher levels of social support. The scale was administered at 20, 28, 36 weeks’ gestation and at 6 weeks postnatal.

Satisfaction with antenatal care was assessed using the Six Simple Questions satisfaction scale (SSQ). The scale was developed specifically for use in the perinatal period, to provide data on the issues that are most likely to impact on women’s perception of the care they receive [34]. It has been shown to reflect changes in satisfaction levels over time, which was a requirement for the present study. Each question has seven possible responses ranging from a score of one (corresponding to ‘strongly disagree’) to a score of seven (corresponding to ‘strongly agree’). Total scores range from 6 to 42, with a higher score indicating a greater level of satisfaction with care. The SSQ was administered at 36 weeks’ gestation and at 6 weeks postnatal.

The following major clinical outcomes were measured: Pregnancy induced hypertension (PIH) which was defined as a diastolic BP ≥90 mmHg and/or a systolic blood pressure ≥140 mmHg on at least two occasions 4 hours apart [35]. PE was defined as PIH and proteinuria of ≥ 2+ measured by urine dipstick and/or ≥ 300 mg/day on 24 hour urine collection [35]. PE resulting in delivery at less than 34 weeks gestation was classified as severe PE. Small for gestational age (SGA) infants were classified in the following two categories based on birthweight for sex and gestational age according to local population standards: 5th-10th percentile and < 5th percentile [36].

Based on a standard deviation (SD) of 2.7 visits [3,4], a sample size of 196 women in each group would give a 90% power to detect a difference in the mean number of visits of 1.0 assuming a type 1 error rate of 2.5%. Anticipating a 30% attrition rate (due to participant withdrawal and failure to retrieve hospital and hand-held notes), 840 participants were required. An intention to treat analysis was performed. Statistical analysis was undertaken using SPSS. For normally distributed data, a one-way ANOVA was conducted to compare means; data are reported as mean and standard deviation (SD). In the case of non-normally distributed data, an independent samples Kruskal-Wallis test was performed when three groups were compared and a Mann–Whitney test was used when two groups were compared; data are presented as median and the interquartile range (IQR). Categorical variables were compared by crosstabulation using Pearson’s Chi squared test and Fisher’s exact test for small samples. The significance level was set at 2.5% to allow for multiple comparisons between groups.

## Results

**Figure 1:**
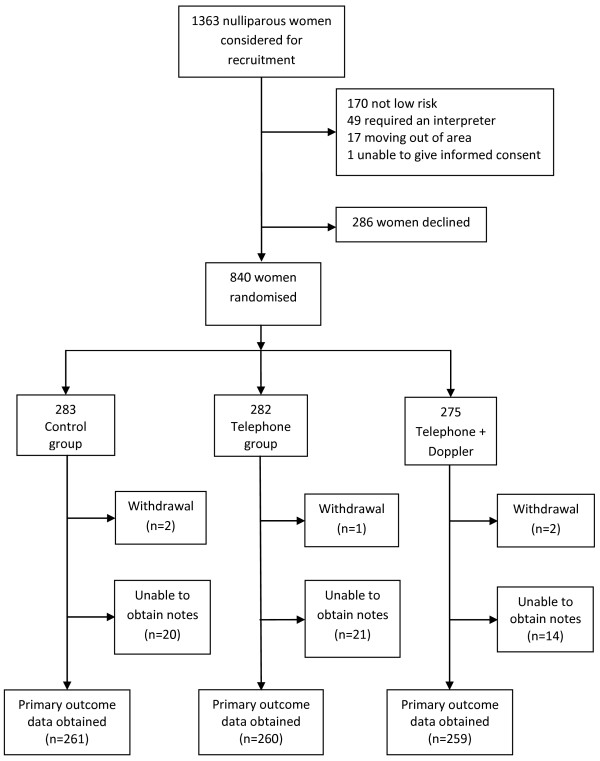
Trial flow chart.

During the trial recruitment phase, a total of 1363 nulliparous women attended for their 20 week ultrasound scan and were considered for inclusion (Figure 1). Of these, 237 (17.3%) women were ineligible: 170 (71.7%) women were not low risk as defined by the NICE antenatal guidelines [1]; 49 (20.6%) required an interpreter; 17 (7.1%) women planned to move out of the area/country during their pregnancy; and one woman (0.4%) had learning disabilities which precluded informed consent. Of the remaining 1126 women who were approached to take part in the trial, 840 (75%) women consented. The reasons stated for not wishing to take part in the trial were as follows: 226 (79%) women did not want to be involved in research; 34 (12%) women felt that they may be worried by the extra information provided by the UADS; 11 (3.8%) women didn’t want any additional support and 15 (5.2%) women were too busy to commit to completing questionnaires or receiving the TSI.

Primary outcome data was not obtained in 60 (7.1%) women due to an inability to obtain the women’s hospital and/or handheld notes because of misfiling. Five women chose to withdraw from the study; one woman stated she disliked answering the items in the questionnaires, one woman withdrew after receiving a risk positive uterine artery Doppler result and the remaining three women did not give a reason.

Baseline characteristics of participants are shown in Table 1. For each characteristic the differences between the three trial groups were small; the groups appear to be well balanced at baseline.

**Table 1:** Baseline characteristics

**C = Control; T = Telephone intervention; T + D = Telephone + Doppler intervention**				
		**Group**		
		**C n = 283**	**T n = 282**	**T + D n = 275**
**Age at recruitment**	**Mean (SD) years**	27.4 (5.8)	26.9 (5.4)	27.8 (5.8)
**Married/Co-habiting**	**n (%)**	237(83.7)	236 (83.7)	230 (83.6)
**White**		258(91.2)	262 (92.9)	262 (93.1)
**Highest educational attainment**	**None**	14 (5.6)	18 (6.9)	13 (4.9)
	**GCSE**^**1**^	73 (29.0)	64 (24.5)	76 (28.7)
	**A level**^**2**^	64 (25.4)	69 (26.4)	69 (26.0)
	**First degree**	72 (28.6)	86 (33.0)	82 (30.9)
	**Higher degree**	29 (11.5)	24 (9.2)	25 (9.4)
	**No response**	31	21	10
**STAI-Trait score**	**Mean (SD)**	36.6(8.9)	35.7 (9.0)	36.3 (8.9)
	**95%CI**	35.4-37.8	34.5-36.9	35.2-37.4
	**No response**	62	61	37

^1^A public examination in specified subjects usually taken by 16 year old schoolchildren; ^2^A public examination in specified subjects usually taken when 17–18 years old.

The proportion of participants who received the TSI at each time point was similar in the T and T + D groups (Table 2). Fewer women received the TSI in both groups at 37 weeks gestation. Uterine artery Doppler waveforms were successfully recorded for all but one woman (1/275); this occurred because the women felt unwell and requested cessation of the ultrasound examination. Of the 274 women who had screening at 20 weeks gestation, 37 (13.5%) had an abnormal result. One woman who received a positive result at 20 weeks gestation withdrew from the study prior to the 24 week follow up scan. Of the 36 women who had a repeat Doppler, five (13.8%) women had a persistently abnormal uterine artery Doppler.

**Table 2:** Number of women who received TSI at each time point

		**Group**			
		**T n =282 (%)**	**T + D n = 275 (%)**	**95% CI**	**p value**
**Antenatal time points**	**29 weeks**	168 (59.5)	167 (60.7)	-0.06-0.09	0.78
	**33 weeks**	170 (60.2)	166 (60.3)	-0.08-0.08	0.98
	**37 weeks**	150 (53.5)	143 (52.0)	-0.09-0.07	0.77

The differences between the groups in the total number of antenatal visits received after 20 weeks gestation were small and not statistically significant (p = 0.74); the numbers of routine and unscheduled visits were also similar in the three groups (Table 3).

**Table 3:** Number of routine and unscheduled antenatal visits after 20 weeks gestation

**Variable**	**C n = 261**	**T n = 260**	**T + D n = 259**
**Median unscheduled visits (IQR)**	2.0 (1.0-4.0)	2.0 (1.0-4.0)	2.0 (1.0-3.0)
**Median routine visits (IQR)**	7.0 (6.0-7.0)	7.0 (6.0-7.0)	6.0 (6.0-7.0)
**Mean total visits (SD)**	8.7 (2.7)	8.9 (3.2)	8.8 (2.9)

The majority of unscheduled visits took place in the hospital Maternity Assessment Unit (MAU) with the community midwife being the next most frequently accessed care provider. The most frequently stated reasons for accessing additional antenatal care, as determined from midwives documentation in the women’s notes were: possible onset of labour which was subsequently not confirmed (14.5%), raised blood pressure (12.3%) and reduced fetal movements (10.2%) (Additional file 1: Table S1).

Only a small number of women received no routine antenatal visits at all (n = 5; 0.64%); this occurred because the women had a very preterm delivery or they developed a significant pregnancy complication. Around a fifth of women in each group received five or fewer routine antenatal visits (C = 19.8%; T = 19.0%; T + D = 21.1%) while slightly more than one fifth received eight routine antenatal visits (C = 23.7%; T = 24.3%; T + D = 20.7%) as a consequence of requiring a post dates review by their midwife. Women who received a screen positive result at 20 weeks accessed the same number of visits (routine: 7; unscheduled: 2) when compared to the rest of the study population.

Differences between the three groups in the level of state anxiety as measured by the STAI at each of the time points were very small and not statistically significant (Table 4). Similarly there were no clinically important differences in the reported levels of social support between the three groups at any of the antenatal time points or at six weeks postnatally. Additionally, there was no significant difference in the STAI scores at 28 (38.5) and 36 weeks (35.7) for women who had a screen positive UADS result at 20 weeks when compared to the scores of the remaining trial participants (28 weeks: 36.5; 36 weeks: 35.6).

**Table 4:** Mean STAI state subscale scores

**Time point**	**Group**	**n**	**Mean score (SD)**	**95% CI**	**F**	**p value**
**20 wks**	**C**	216	36.2 (10.5)	34.8-37.6		0.48
	**T**	217	36.9 (10.9)	35.4-38.3	0.72	
	**T + D**	236	35.7 (10.0)	34.4-37.0		
**28 wks**	**C**	194	36.5 (11.0)	34.9-38.0		0.38
	**T**	181	35.9 (10.5)	34.3-37.4	0.94	
	**T + D**	189	37.4 (11.2)	35.8-39.0		
**36 wks**	**C**	166	36.7 (10.9)	35.0-38.4		0.68
	**T**	159	37.1 (10.3)	35.5-38.8	0.37	
	**T + D**	170	36.2 (9.9)	34.7-37.7		
**6 wks PN**	**C**	128	32.5 (9.6)	30.8-34.2		0.66
	**T**	151	31.6 (8.4)	29.9-33.2	0.41	
	**T + D**	162	31.9 (9.4)	31.0-32.8		

The levels of social support did not alter during pregnancy or six weeks after birth with mean scores indicating high levels of support throughout the women’s involvement in the trial (Additional file 2: Table S2). There were no statistically significant differences in levels of satisfaction between the three trial groups. The median scores suggest high levels of satisfaction with antenatal care across all three trial groups when measured at six weeks postnatally (C = 35.5, T = 28.0, T + D = 29.0, p = 0.27) (Additional file 3: Table S3). Questionnaire response rates at each time point are shown in Additional file 4: Table S4.

Clinical outcomes are shown in Table 5, although it is accepted that the study was not powered to detect differences between the groups for clinical outcomes. Three out of five women who were screen positive at 24 weeks gestation delivered SGA infants and two of these women also had PIH. There were no clinically significant differences between groups in the proportion of women who had a SGA baby (BW ≤ 10th centile) in the group of women who had negative UADS at 20 weeks compared to those who had a positive result at 20 weeks and then a negative result at 24 weeks (p = 0.14). More women with positive UADS at 20 and 24 weeks delivered SGA infants compared to women who had negative UADS at 20 or 24 weeks’ gestation (p = 0.003). Because the number of women with a positive result was so few, prediction statistics were not calculated. One woman experienced a miscarriage at 23 weeks’ gestation and one participant opted for termination of pregnancy due to fetal abnormality. Two pregnancies ended in stillbirth, one in the control group and one in the telephone intervention group. There was no difference between the groups in the prevalence of hypertensive disorders, mode of delivery or delivery-related complications.

**Table 5:** Maternal and infant outcomes

		**Group**				
		**C (n = 278)**		**T (n = 276)**	**T + D (n = 268)**	**X**^**2**^	**p**
**Gestation at delivery (days)**	**Mean (SD)**	279.3 (16.0)	279.9 (14.9)	279.3 (13.4)	2.59	0.30
**Onset of labour**	**Spontaneous**	236 (84.9)	221 (80.1)	211 (78.7)		
	**Induced**	34 (12.2)	48 (17.4)	50 (18.7)	4.74	0.31
	**No labour**	8 (2.9)	7 (2.5)	7 (2.6)		
**Reason for induction**	**Postdates**	21 (61.8)	29 (60.4)	24 (48.0)		
	**Preeclampsia**	6 (17.6)	8 (17.0)	9 (18.0)	4.06	0.67
	**Fetal concern**	4 (11.7)	3 (6.4)	6 (12.0)		
	**Other**	3 (8.8)	8 (16.7)	11 (22.0)		
**Mode of delivery**	**Normal vaginal**	121 (43.5)	114 (41.3)	113 (42.2)		
	**Assisted vaginal**	97 (34.9)	92 (33.3)	90 (33.6)	1.53	0.95
	Emergency LSCS^**^	52 (18.7)	63 (22.8)	58 (21.6)		
	**Elective LSCS**	8 (2.9)	7 (2.5)	7 (2.6)		
**Delivery related complication**	**PPH (> 500mls)**	36 (21.6)	32 (19.2)	39 (23.4)		
	**Shoulder dystocia**	4 (2.4)	4 (2.4)	5 (3.0)	2.54	0.86
	**3rd/4th degree tear**	9 (5.4)	12 (7.2)	8 (4.8)		
**Infant outcome**	**Live birth**	274 (99.3)	274 (99.6)	269 (99.6)		
	**Stillbirth**	1 (0.4)	1 (0.4)	0 (0.0)	1.99	0.73
	**Miscarriage/TOP**^**#**^	1 (0.4)	0 (0.0)	1 (0.4)		
**Infant sex**	**Male**	138 (50.2)	141 (51.3)	132 (49.3)		
	**Female**	137 (49.8)	134 (48.7)	136 (50.7)		
**Birthweight (grams)**	**Mean (SD)**	3395 (530.7)	3346 (555.9)	3356 (546.7)	0.60	0.55
**Small for gestational age**	**5th-10th percentile**	12 (4.4)	15 (5.5)	13 (4.9)	0.72	0.94
	**<5th percentile**	12 (4.4)	10 (3.6)	9 (3.4)		
**Preterm < 37 weeks**		9 (3.2)	17 (6.2)	14 (5.2)	2.66	0.26
**Congenital abnormality**		7 (2.5)		5 (1.8)	2 (0.7)		
Admission to SCBU^*^		12 (4.3)	9 (3.3)	8 (3.0)	0.89	0.64	
**Severe pre eclampsia**		0 (0.0)	1 (0.4)	0 (0.0)			
**Pre eclampsia**		6 (2.2)	7 (2.2)	6 (2.6)	7.8	0.45	
**Pregnancy induced hypertension**		28 (10.1)	18 (6.5)	24 (8.9)			
**Pregnancy induced proteinuria**		0 (0.0)	3 (1.1)	1 (0.4)			

SCBU* - Special care baby unit; LCSC** - Lower section Caesarean section; TOP^#^ - Termination of pregnancy.

## Discussion

The results showed that the support interventions tested did not affect the total number of antenatal visits that women required and there was no difference between the trial groups for any of the psychological outcomes measured. All of the women in the study were able to provide at least one contact telephone number showing that access to the TSI was universal for this population.

The results demonstrated that there are significant challenges to the implementation of a TSI, with most women receiving fewer calls than planned. The telephone intervention was successfully delivered to 59% of women at 29 and 33 weeks’ gestation, decreasing to 52% of women at 37 weeks. The mean number of telephone calls received by women was 1.6, which is just over half the three calls that were proposed. The reduction in the success rate of the calls at 37 weeks’ gestation may be due to women having moved house and/or changed telephone numbers while 7% had given birth. Prior studies of TSI during pregnancy and the postpartum period have experienced similar difficulties in implementing the intervention [17,37,38]. It is possible that the women who were not reached were most in need of support although there was no correlation between the number of calls received and the STAI score at 20 weeks gestation (r = 0.04, p = 0.38) or between the number of calls and the number of unscheduled antenatal visits that women made (r = 0.23, p = 0.61).

The introduction of UADS into the clinical service was straightforward with measurements obtained in 99.6% of cases; this is consistent with a previous study [39]. The screen positive rate at 20 weeks was comparable to previous studies of UADS in unselected populations (9-16%) [39,40]. However the rate at 24 weeks (1.8%) was lower than the 3.9% rate reported by Harrington et al. [40], using a similar definition of abnormal, and the 5.1% rate reported by Bower al [39] based on a resistance index (RI) > 95th percentile and any notching. One study in ‘low risk’ nulliparous women reported much higher screen positive rates (based on mean RI > 0.58) at 20 and 24 weeks (30.0% and 8.6% respectively) [41]. Overall 8.7% of the study population delivered an SGA infant and the rate was higher in women with abnormal UADS. The prevalence of PE (2.3%) was consistent with previous studies employing the same definition [42,43].

Women in the present study demonstrated expected levels of anxiety prior to the commencement of the interventions with mean STAI-state scores at 20 weeks gestation of 36–37. These results are comparable with other studies of similar samples of women during pregnancy [44-48]. There was no significant difference in the STAI scores at 28 (38.5) and 36 weeks (35.7) for women who had a positive UADS result at 20 weeks, when compared to the scores of the remaining trial participants (28 weeks: 36.5; 36 weeks: 35.6). Furthermore, those who received a positive UADS result accessed the same number of antenatal visits (routine: 7; unscheduled: 2), when compared to the rest of the study population. These findings demonstrate that being deemed at increased risk of placental-mediated disorders did not result in women accessing additional antenatal visits or having increased levels of anxiety.

Overall, participants’ anxiety levels remained stable over the duration of the pregnancy, which is consistent with a previous study using the same scale [44]. It is reassuring to find that this population is not highly anxious in general, although it is recognised that anxiety is only one component of a woman’s psychological experience of her first pregnancy.

The total number of antenatal visits that women accessed was perceived to be an important outcome given that prior trials of a reduced visit schedule in developed countries had failed to implement the intervention with women consistently having more visits than recommended [3,4,6]. The decision to power the study to detect a difference of one antenatal visit was made because of the potential economic implications of an increase or decrease of one visit per woman when considered on a national scale; figures published by the Department of Health for 2012–2013 show that the mean cost of an antenatal visit to a community midwife was £51 (range £38 - £58) [49], and there were 729,674 live births in England and Wales in 2012 [50].

It was not clear from prior trials why women make unscheduled antenatal visits and hence what proportion of these visits could potentially be avoided by the provision of an effective support intervention [51,52]. This study identified that nearly 80% of women had at least one additional visit and 67% of additional visits were made because of pregnancy complications detected by themselves or by their midwife (e.g. hypertension, reduced fetal movements, vaginal bleeding), that require a face-to-face consultation. These findings clearly demonstrate that a significant proportion of low risk nulliparous women will experience complications that cannot be predicted by the current selection criteria and are not necessarily amenable to support interventions [26].

The relationship between physical ill health and psychological wellbeing is complex. Although the majority of presentations for additional care are cited as being for physical problems, it is likely that a proportion of pregnancy symptoms and complications may be the manifestation of a lack of social support, social difficulties or increased anxiety and worry. This could result in an increased number of unscheduled visits to health professionals. Research in non-pregnant patients has shown that frequent attendance in general practice is correlated with psychological distress and social factors such as unemployment and single status [53].

A study using a discrete choice experiment showed that women did place value on the addition of UADS and were willing to reduce the number of routine visits they received and pay £302 to have the screening incorporated into their care [54]. However the possible negative psychological consequences of introducing screening in a low risk population are important. Although the findings suggest that UADS does not increase anxiety in low risk nulliparous women, the number of screen positive women in the study was very low. It is worth noting that some women declined to take part in the study because they were concerned about receiving worrying information from UADS (12% of those who declined). Thus we are unable to draw any conclusions about the impact of receiving a screen positive result and being deemed at higher risk for developing PE and/or SGA.

This is the first study to evaluate interventions designed to provide low risk nulliparous women with additional support and reassurance during pregnancy, following the implementation of a reduction in the number of routine antenatal visits [1]. The study provides novel information about the reasons why women make unscheduled antenatal visits; most unscheduled visits are the result of symptoms and concerns that are unlikely to be ameliorated by a support intervention [52].

There are a number of recognised limitations to the study. The exclusion of women who required an interpreter means that the sample is not representative of the entire nulliparous population or generalisable to the wider population. A further limitation of the study was the exclusion of some teenage women. At the time of recruitment, all women under 18 years living in Newcastle were provided with additional support from the teenage pregnancy team.

Although the results of the study showed that the telephone intervention calls conferred no statistically significant benefit to women in terms of the number of antenatal visits, levels of anxiety or social support, it remains a possibility that a proportion of women would find telephone support useful; women participating in a discrete choice experiment of components of antenatal care were willing to pay the greatest sum (£368) to be provided with a TSI in addition to their usual antenatal care [54]. A recent review of TSIs introduced during pregnancy and six weeks after birth, reported that outcomes were encouraging but inconsistent [14]. Further research is required to determine whether telephone intervention are economically viable and have the potential improve women’s experiences and outcomes.

## Conclusions

Provision of additional telephone support (with or without UADS) in low risk nulliparous women did not reduce the number of unscheduled antenatal visits or reduce anxiety. The majority of low risk nulliparous women require face-to-face antenatal visits in addition to the recommended schedule of care. This study provides some evidence to explain where woman present for unscheduled antenatal care and the reasons that prompt them to do so.

Alternative methods of contacting pregnant women offers healthcare providers with opportunities to redirect resource use while providing the flexible care that women value. Future research should focus on studies that investigate innovative provision of care by utilising new technologies, alternative care settings and organisation of care with an aim to provide a service that is responsive, progressive and woman-centred.

### Details of ethics approval

Ethical approval was obtained from the Joint Ethics Committee of the Newcastle and North Tyneside Health (ref no: 2003 /208).

## Abbreviations

ANOVA: Analysis of variance; CI: Confidence interval; GCSE: General certificate of secondary education; IQR: Interquartile range; LR: Likelihood ratio; LSCS: Lower section Caesarean section; MAU: Maternity assessment unit; NCCWCH: National Co-ordinating Centre for Women’s and Children’s Health; NICE: National Institute of Clinical Excellence; PE: Pre-eclampsia; PI: Pulsatility index; PIH: Pregnancy induced hypertension; PPH: Postpartum haemorrhage; SCBU: Special care baby unit; SD: Standard deviation; SGA: Small for gestation age; SSQ: Six simple questions; STAI: State-trait anxiety inventory; TOP: Termination of pregnancy; TSI: Telephone support intervention; UADS: Uterine artery Doppler screening; GP: General practitioner; UK: United Kingdom; mmHg: Milligrams of mercury; X2: Chi square.

## Competing interests

The authors declare that they have no competing interests.

## Authors’ contributions

VS, JH and SR had roles in the conception of the study; all of the authors were involved in the initial design. VS was responsible for the coordination of the study, the analysis of data, the first draft and revisions and agreed the final version. INS provided statistical advice and contributed to the data analysis. JH and SR contributed to the data analysis, revisions to the draft and agreed the final version. All authors read and approved the final manuscript.

## Pre-publication history

The pre-publication history for this paper can be accessed here:

http://www.biomedcentral.com/1471-2393/14/121/prepub

## Supplementary Material

Additional file 1: Table S1 Location and reason stated for unscheduled antenatal visits after 20 weeks gestation. Click here for file

Additional file 2: Table S2 Total DUFSS scores. Click here for file

Additional file 3: Table S3 Comparison of total SSQ scores at 36 weeks gestation. Click here for file

Additional file 4: Table S4 Questionnaire response rate. Click here for file
