# Supplementary material for: Antenatal telephone support intervention with and without uterine artery Doppler screening for low risk nulliparous women: a randomised controlled trial
Source: BMC Pregnancy Childbirth. 2014 Mar 31;14:121. doi: 10.1186/1471-2393-14-121 (PMC4021157; doi:10.1186/1471-2393-14-121)
Supplement: Additional file 1: Table S1 — Location and reason stated for unscheduled antenatal visits after 20 weeks gestation. [file 1471-2393-14-121-S1.pdf]

Supplementary table 1 - Location and reason stated for unscheduled antenatal visits after 20 weeks gestation

		**Group**		
		**C**	**T**	**T+D**
		**n=591**<br>n (%)	**n=667**<br>n (%)	**n=650**<br>n (%)
**Location of visit**	Maternity Assessment Unit	326(55.2)	395(59.2)	391(60.2)
	Community midwife	177(29.9)	143(21.4)	159(24.5)
	General practitioner	4(0.7)	7(1.0)	4(0.6)
	Hospital antenatal clinic	46(7.8)	71(10.6)	53(8.2)
	Home	17(2.9)	15 (2.2)	23(3.5)
	Other	21(3.6)	36 (5.4)	19(2.9)
**Reason for visit**	Reduced fetal movements	72(12.2)	59(8.8)	66(10.2)
	Raised blood pressure	81(13.7)	58(8.7)	96(14.8)
	Premature rupture of membranes	12(2.0)	24(3.6)	17(2.6)
	Unwell	17(2.9)	9(1.3)	18(2.8)
	Vaginal bleeding	19(3.2)	47(7.0)	34(5.2)
	Itching/ Obstetric Cholestasis	19(3.2)	31(4.6)	21(3.2)
	? Onset of labour	89(15.1)	81(12.1)	100(15.4)
	Abdominal pain	18(3.0)	34(5.1)	22(3.4)
	Breech/External cephalic version	37(6.3)	28(4.2)	36(5.5)
	Preterm labour	1(0.2)	6(0.9)	7(1.1)
	Suspected SGA* fetus	10(1.7)	27(4.0)	23(3.5)
	Monitoring of SGA* fetus	5(0.8)	49(7.3)	7(1.1)
	Other	88(14.9)	97(14.5)	91(14.0)
	No reason specified	123(20.8)	117(17.5)	111(17.1)

SGA* - Small for gestational age
