# Supplementary material for: Antenatal telephone support intervention with and without uterine artery Doppler screening for low risk nulliparous women: a randomised controlled trial
Source: BMC Pregnancy Childbirth. 2014 Mar 31;14:121. doi: 10.1186/1471-2393-14-121 (PMC4021157; doi:10.1186/1471-2393-14-121)
Supplement: Additional file 2: Table S2 — Total DUFSS scores. [file 1471-2393-14-121-S2.pdf]

Supplementary table 2 - Total DUFSS scores

Time point	Group	n	Median (IQR)	X^2	p value
20 wks	C	227	11 (7.0)		
	T	229	10 (6.0)	0.75	0.68
	T+D	244	11 (6.7)		
28 wks	C	196	10 (7.0)		
	T	191	11 (6.0)	2.21	0.33
	T+D	196	11 (8.0)		
36 wks	C	172	10 (6.0)		
	T	167	10 (7.0)	2.00	0.36
	T+D	171	11 (7.0)		
6 weeks postnatal	C	140	10 (8.0)		
	T	161	11 (7.0)	0.15	0.92
	T+D	175	11 (7.0)		
