# Supplementary material for: Antenatal telephone support intervention with and without uterine artery Doppler screening for low risk nulliparous women: a randomised controlled trial
Source: BMC Pregnancy Childbirth. 2014 Mar 31;14:121. doi: 10.1186/1471-2393-14-121 (PMC4021157; doi:10.1186/1471-2393-14-121)
Supplement: Additional file 3: Table S3 — Comparison of total SSQ scores at 36 weeks gestation. [file 1471-2393-14-121-S3.pdf]

Supplementary table 3: Comparison of total SSQ scores at 36 weeks gestation

Time point	Group	n	Median (IQR)	X^2	p value
**36 wks**	C	109	29.0 (6.5)		
	T	110	28.0 (5.2)	2.55	0.27
	T+D	116	29.0 (6.0)		
**Postnatal**	C	134	35.5 (9.0)		
	T	139	35.0 (8.0)	1.30	0.52
	T+D	154	35.0 (9.0)		
