# Supplementary material for: Antenatal telephone support intervention with and without uterine artery Doppler screening for low risk nulliparous women: a randomised controlled trial
Source: BMC Pregnancy Childbirth. 2014 Mar 31;14:121. doi: 10.1186/1471-2393-14-121 (PMC4021157; doi:10.1186/1471-2393-14-121)
Supplement: Additional file 4: Table S4 — Questionnaire response rate. [file 1471-2393-14-121-S4.pdf]

Supplementary table 4 - Questionnaire response rate

		**Group**				
		**C**	**T**	**T+D**	**X^2**	
Time points	**Total**	n =282	n =283	n =275		**p value**
	**20 weeks**	232 (82.2)	229 (80.9)	242 (88.0)	5.75	0.06
	**28 weeks**	199 (70.5)	193 (68.1)	194 (70.5)	0.49	0.99
	**36 weeks**	173 (61.3)	168 (59.3)	175 (63.6)	1.07	0.58
	**6 weeks postnatal**	131 (46.4)	166 (58.6)	155 (56.3)	9.53	0.001
